# *Oxya hyla hyla* (Orthoptera: Acrididae) as an Alternative Protein Source for Japanese Quail

**DOI:** 10.1155/2014/269810

**Published:** 2014-10-29

**Authors:** Mousumi Das, Suman Kalyan Mandal

**Affiliations:** ^1^Entomology Research Unit, Department of Zoology, Visva-Bharati University, Santiniketan, West Bengal 731235, India; ^2^Department of Botany, Visva-Bharati University, Santiniketan, West Bengal 731235, India

## Abstract

Nutrient composition of the grasshoppers *Oxya hyla hyla* showed that they are a rich nutrient source containing 687.7 g protein/kg of dry body weight. Their antinutrient values fell within nutritionally acceptable values of the poultry bird *Coturnix japonica japonica* (Japanese quail). The most required essential amino acids and fatty acids were also present in sufficient amount. For feeding trial nine diets were formulated on an equal crude protein (230 g/kg) basis with grasshopper meal, fish meal, and soybean meal. Three sets of diets with grasshopper meal were prepared with 50 g/kg, 100 g/kg, and 150 g/kg grasshopper of total feed. Similarly, other diet sets were prepared with fish meal and also with soybean meal. Results were compared with another group of Japanese quails fed on a reference diet that was considered as control. Two experiments were conducted with a total number of 600, seven-day-old, Japanese quails. In experiment 1 for determination of growth performance, quails were randomly distributed into ten groups of males and ten groups of females containing 30 birds each. In experiment 2 for determination of laying performance, identical ten groups were prepared in ten repetitions (2 females and 1 male in each group) from the six-week-old birds of experiment 1. Birds of diet set GM2 have gained the highest body weight (male 4.04 g/bird/day; female 5.01 g/bird/day) followed by birds of FM3 diet set (male 3.72 g/bird/day; female 4.40 g/bird/day), whereas birds of reference diet have gained 3.05 g/bird/day for male and 3.23 g/bird/day for female. Feed conversion ratio (FCR) of birds fed with GM2 was the lowest (male 3.33; female 2.97) whereas FCR of R group was higher (male 4.37; female 4.65) than grasshopper meal and fish meal based diets. Hen day production percentage was higher (72.2) in GM2 group, followed by FM3 (63.5) group. R group had lower 1st egg weight (9.0 g), weight gain (8.2 g), percentage of hen day production (41.8%), higher feed intake (33.6 g/day/bird), and age at 1st laid egg than the grasshopper meal and fish meal based diets. So growth and laying performance of the birds were significantly better in grasshopper meal and fish meal added diet fed sets than the reference diet fed group; among all the dietary groups 100 g/kg grasshopper meal added diet mostly gave significantly better results followed by 150 g/kg fish meal added diets. It was ascertained that the *O. hyla hyla* meal had pronounced positive response on the birds. So, the quails could be easily fed 100 g/kg grasshopper meal added diet as it was the most suitable alternative feedstuff compared to the conventional protein source based diets.

## 1. Introduction

Over one billion people in developing countries are suffering from malnutrition and protein deficiency [1, 2] that are closely associated with poverty. Nowadays, poverty reduction largely focuses on poultry farming [3]. Poultry meat contributes approximately 370 g/kg to the total animal protein supply and there is a possibility of growth and expansion of this industry [4]. However, the progress made so far in the poultry sector is currently being undetermined by the escalating cost of their feeds, increasing competitive demand for them by man and animals, and scarcity of the conventional animal protein source, that is, fish meal, and conventional plant protein source, that is, grains [5]. Therefore, to reduce the feed cost, which accounts for 75% to 80% of the total cost of production [6], research efforts are being geared towards evaluating alternative, good quality renewable protein sources that can replace or substitute scarce, expensive, and elusive conventional protein sources used in poultry nutrition. One of such alternatives is insects which represent a significant biomass that is still not fully utilised around the world. Many studies have shown that insects contain large quantity of protein of good quality [7] and high digestibility [8]. They are also good sources of fat, essential fatty acids and amino acids, vitamins, and minerals [8, 9]. Among the edible insects, acridids are known to have a high nutritional value (protein content 521 g/kg to 771 g/kg) [7] and are found in high density in various parts of the world. So, they might be harvested and could be mass reared under controlled conditions for utilization of this nutrient resource.

According to de Conconi et al., insects have played an important role in poultry nutrition [10]. Studier and Sevick published that insects fulfil the nutritional requirements for growth and reproduction in birds [11]. Ramos-Elorduy and DeFoliart et al. estimated that acridids have a high nutritional value and can be used to formulate good quality feed for livestock [7, 12]. In this regard, Wang et al. formulated high protein diets with the acridid* Acrida cinerea* and proved that it is an acceptable feed for broiler without any adverse effect [13]. Therefore, a study was conducted on nutritional profile and protein quality of* O. hyla hyla* (Serville) for evaluating their potential as a nonconventional protein source in the diet of Japanese quails (*Coturnix japonica japonica*), in comparison with conventional animal protein source, that is, fish meal, and conventional plant protein source, that is, soybean meal, with special reference to the performance characteristics and overall health status of growing and laying quails. The main focus of the present study was to use grasshopper meal as supplement in the formulated diets at different proportions and to find out which proportion of grasshopper meal could be best for the selected poultry bird. Next, it was also evaluated whether the results are comparable with other formulated diets having conventional plant protein source and animal protein source to establish the suitability of grasshopper meal as an alternative protein source for poultry.

## 2. Materials and Methods 

### 2.1. Sample Collection and Preparation

Samples of selected adult grasshoppers were collected from nearby agricultural and grassland fields of Santiniketan (23°39′N, 87°42′E), Birbhum, West Bengal, India, by sweeping technique. Shortly after collection, samples were stored at −20°C until required for analysis. Before analyses, samples were oven-dried at 60°–70°C for 72 h and crushed through a 60-mesh screen.

### 2.2. Method of Chemical Analysis

Moisture, ash, and fat contents were estimated by AOAC methods 934.01, 942.05, and 920.39, respectively, where the protein content (N × 6.25) was determined by Kjeldahl method (984.13) [14]. Carbohydrate was obtained by difference (100 − sum of moisture, protein, fat, and ash). Amino acid content of the sample was determined by the PICO.TAG method with few modifications [15]. Samples were acid hydrolised with hydrochloric acid (6 mol/L) at 110°C for 24 h before analysis. Fatty acid contents were determined with an Agilent 6890 GC (PA, USA) [16]. Mineral contents were determined with a Buck Atomic Absorption Spectrophotometer (Buck Scientific Inc., Norwalk, CT, USA). Vitamins B1, B2, and B3 were determined by HPLC using the method described by Wimalasiri and Wills [17]. Vitamin C was estimated using acetic acid and metaphosphoric acid, followed by determination with HPLC using fluorescence [18]. Alkaline saponification of the sample involved elimination of fats, liberation of natural retinol in the cells, and hydrolysis of added Vitamin A. Vitamin A was determined by HPLC and detected using UV and fluorescence [19]. Tannin, phytic acid, and oxalate contents of the dried sample were determined by the standard methods [20–22].

### 2.3. Experimental Diets

Nine isonitrogenous (230 g/kg) diets were formulated with the feed ingredients and diet 10 was used as a reference diet that was formulated in the identical proportion of a locally available market feed, and with this other diets were compared. Three sets of diets with grasshopper meal as nonconventional protein source were prepared with 50 g/kg, 100 g/kg, and 150 g/kg grasshopper of total feed. Similarly, another three diet sets were prepared with conventional animal protein source, that is, fish meal, and three diets sets also with conventional plant protein source, that is, soybean meal. In case of fish meal based diets* Gudusia chapra* were used in oven-dried powder form, and in soybean meal based diets, thin-layer toasting (30 mm) of soybean seeds at 130°C for 10 min was used to inactivate trypsin inhibitor [23]. Regarding the feed formulation in all the formulated diets the amount of maize was fixed. The amounts of other two ingredients, that is, rice husk and groundnut cake, of the formulated diets were mixed in such a way that each of the formulated diets contains about 230 g/kg crude protein. Maintaining the amount of protein, adjustment of the proportion of these two ingredients was done by Pearson's square method.  Table 2 shows the composition of the diet to nourish the quails according to NCR requirements [24]. Composition of formulated diets, which were supplied to the growing quails of experiment 1 and laying quails of experiment 2, were the same except the vitamin-mineral mixture composition part. In case of laying quails of experiment 2, calcium content of diet covers the recommended requirements [24] by increasing the level of calcium content of vitamin-mineral mixture. Proximate compositions of the formulated feeds were analyzed according to AOAC method.

### 2.4. Bird Husbandry and Experimental Design

A total number of 600, seven-day-old, Japanese quail birds (300 males and 300 females) were obtained from Ghosh enterprise, Kolkata, India, which belonged to the same breed. Two experiments were conducted to determine the growth performance, egg laying performance, egg quality, and overall health status of Japanese quails. Quails were housed at 24-hour lighting for the first three weeks and in the following weeks at a 16 : 8 light : dark cycle [25]. The temperature started at 36°C and it was decreased by 3°C every week and fixed at 24°C after 4 weeks of age [25].

#### 2.4.1. Experiment 1

*Experimental Design.* The experimental quails were randomly distributed into ten groups of males and ten groups of females containing 30 birds each. Each group contained 3 replicates of 10 quails. All the three replicates of ten groups like GM1 (50 g grasshopper/kg of total feed), GM2 (100 g grasshopper/kg of total feed), GM3 (150 g grasshopper/kg of total feed), FM1 (50 g fish meal/kg of total feed), FM2 (100 g fish meal/kg of total feed), FM3 (150 g fish meal/kg of total feed), SM1 (50 g soybean meal/kg of total feed), SM2 (100 g soybean meal/kg of total feed), SM3 (150 g soybean meal/kg of total feed), and reference diet (R) were kept in California-type battery cage which provides 200-square-centimetre area per bird. Experimental feed and water were supplied for consumption. Observation period lasted for 6 weeks.

*Data Collection.* At the start of experiment, first of all body weights were taken, followed by subsequent weekly basis. Weekly feed intake and fecal matter excreted were monitored on group basis. Food consumption and utilization was measured with the parameters protein efficiency ratio (PER) and feed conversion ratio (FCR) as suggested by de Silva and Anderson [26].

*Formulae.* Consider the following equations:

(1) $$\begin{array}{c} \text{PER}=\frac{{WW}_{t}}{{DW}_{p}}\\ \text{FCR}=\frac{F}{W_{t}}\\ \text{N}\;\text{retention}=F\times \text{N}1-E\times \text{N}2, \end{array}$$

where  *WW*_*t*_ is wet weight gained; *DW*_*p*_ is dry weight of protein in food; *F* is average dry weight of food ingested per individual; *W*_*t*_ is dry weight gained; N1 is nitrogen content in feed; N2 is nitrogen content in fecal matter; and *E* is fecal matter excreted.

At the end of experiment 1, three males or three females from each group were killed by cervical dislocation and decapitation. Soon after blood samples were collected in heparinized tubes sand blood smears were prepared, air-dried, and stained with 1 part Giemsa: 20 parts distilled water for 20 min prior to fixation in ethyl alcohol for 3 min. Smears were washed in running tap water for 1 min. One hundred leucocytes were counted, and the relative proportions of lymphocytes, neutrophils, eosinophils, basophils, and monocytes were determined. Blood samples containing tubes were kept at 4°C for overnight clotting and then samples were centrifuged for 20 min at 4000 g. The separated serum was transferred to Disease Investigation Veterinary Laboratory, Suri, India, for serum chemistry analysis.

For estimation of meat composition, feathers were plucked from decapitated quails and then carcasses were wrapped individually in polythene bags and immediately placed in a freezer (−20°C). Frozen meat was thawed for 24 h in a refrigerator (+4°C) prior to chemical analysis. Meat was separated from the bone and skin and then mixed thoroughly for homogeneity. The samples of meat were analyzed for moisture, fat, protein, and ash contents according to AOAC methods [14]. Mineral contents were measured using an atomic absorption spectrophotometer (Buck Scientific Inc., Norwalk, CT, USA).

#### 2.4.2. Experiment 2

*Experimental Design.* Identical ten groups were prepared in ten repetitions (2 females and 1 male in each group) from the six-week-old birds of experiment 1. Each group was provided with definite diet for ad libitum consumption. This experiment lasted for ten weeks.

*Data Collection.* During the laying period, age at first egg was noted and weights of such eggs were recorded. Performance characteristics were monitored in terms of weekly % hen day production ([{total  number  of  eggs}/{total  number  of  days × number  of  hens}] × 100), feed intake, and body weight gain.

First ten eggs of each group were assessed through two consecutive days per month for internal and external egg quality traits. For external quality, data on egg weight, egg length, egg breadth, egg shape index, and shell thickness were collected. For internal egg quality, egg proportions were determined. Each egg was carefully broken and shells were separated and air-dried overnight and then they were weighed. Relative weight was calculated by relating the shell weight to the weight of the egg. An egg separator was used to separate the yolk from albumen. Relative yolk and albumen weights were calculated in percentages. Eighteen eggs from each group were divided into 3 replicates of 6 eggs each. Yolks and albumins of 3 eggs from each replicate were separated, while those of the remaining 3 eggs were blended together (i.e., total edible parts of the egg), and their chemical composition was determined according to AOAC method [14].

At the end of experimental period, birds from each group were weighed after fasting for 12 h and slaughtered; their feathers were plucked and the total inedible parts (head, legs, and inedible viscera) were taken aside. Then, the remaining carcass (dressed weight) was weighed. The abdominal fat was removed from the parts around the viscera and gizzard was weighed. The liver, spleen, gizzard, heart, pancreas, intestine, caecum, ovary, and testes were separated and individually weighed, and intestine and caecum lengths were measured. The percentages of carcass yield and organs to live body weight were calculated. Testes volume was measured volumetrically using the Archimedes principles of water displacement in a measuring cylinder. The length and width of each testis was measured using a vernier caliper.

### 2.5. Statistical Analysis

Amino acid, fatty acid, vitamin, mineral, and antinutrient contents of grasshopper meal are expressed as mean ± SD. Results of the feeding trial are expressed as mean values per individual along with their pooled SEM (Tables 4 to 14). All the data were subjected to one-way ANOVA. Where significant differences were found, the means were compared using Duncan's Multiple Range Test.

## 3. Results

### 3.1. Nutrient and Antinutrient Composition of Acridid Meal

The proximate nutritional compositions of grasshopper meal were crude protein (687.7 g/kg), crude fat (73.5 g/kg), crude fibre (66.2 g/kg), carbohydrate (197.3 g/kg), and ash (40.8 g/kg). The result of proximate amino acid, fatty acid, vitamin, mineral composition, and antinutrient content of selected acridid on dry matter basis are listed in Tables 1 and 2. The percentage of total essential amino acid was 592.42 g/kg and nonessential amino acids were 407.74 g/kg. All the essential amino acids were present in the protein portion of the selected acridid with threonine, lysine, histidine, and phenylalanine constituting the major essential amino acids.

Contents of unsaturated fatty acids of the acridid were higher, especially the oleic acid, linoleic acid, and linolenic acid. The total unsaturated fatty acid in the acridid oil was 891.7 g/kg of total crude fat. Total five vitamins, namely, retinol, thiamine, riboflavin, niacin, and ascorbic acid, were detected in the body tissues of the selected acridid. Thiamine was found to be present in very low amount, whereas retinol, riboflavin, niacin, and ascorbic acid were quite high. Total six minerals were estimated. Calcium, magnesium, and iron were the major elements in the acridid. Four antinutritional factors, tannin (condensed), oxalate, phytin bound phosphorus (phytin P), and phytin, were analyzed for the acridid. All of them were found to be present in very low amount.

### 3.2. Experimental Diets

Ingredients and chemical composition of different experimental diets were shown in Table 3. The calcium contents of formulated diets of growing quails and laying quails were 15.1 g/kg (*P* = 0.65) and 25.5 g/kg (*P* = 0.87), respectively.

### 3.3. Experiment 1

Birds in all the dietary groups were fed actively on the experimental diets. There was no rejection of feed until the end of the experiment. The acceptability of the diets was similar. Only in case of SM3 dietary group, one bird died at 4th week of the experiment. Otherwise, no mortality or any sign of disease was observed in any dietary group during the experimental period.

#### 3.3.1. Growth Performance

The feed intake and growth parameters such as body weight gain, feed conversion ratio (FCR), protein efficiency ratio (PER), and nitrogen retention of male bird were presented in Table 4.

The initial weight of 7-day-old birds was almost the same (*P* = 0.86). The body weight gain per day per male growing birds in this study was influenced (*P* < 0.01) by dietary treatments. It is notable that the birds on diet GM2 containing 100 g/kg grasshopper meal gained the highest (*P* < 0.01) weight, compared to those in the other dietary groups, followed by FM3 group of 150 g/kg fish meal, whereas birds on SM3 diet containing 150 g/kg soybean gained the least weight. Diet R (the reference diet) gained nearly the same weight (*P* > 0.05) as in diet FM1 containing 50 g/kg fish meal and SM2 containing 100 g/kg soybean meal. Feed intake of single male bird per day was higher in FM3 group (*P* < 0.01), followed by FM2 dietary group, fed on 100 g/kg fish meal diet. It was lower in SM3 dietary group. There was no significant (*P* > 0.05) difference in feed intake among the birds fed on diet R, 50 g/kg, 100 g/kg, and 150 g/kg grasshopper meal diet, 50 g/kg fish meal diet, and 100 g/kg soybean meal diet. Higher (*P* < 0.01) result of PER was noticed in GM2 dietary group, followed by GM3 group, and the least value was obtained in SM3 dietary group. No significant effect was observed among the birds fed on the rest of the dietary groups. The feed conversion ratio (FCR) of birds fed with GM2 was low (*P* < 0.01) and the birds fed with SM3 was high. The values of FCR had no significant effect among the dietary groups of 50 g/kg and 100 g/kg fish meal and soybean meal diets and R diet. R group had higher mean value of FCR than grasshopper meal and fish meal based diets. Daily nitrogen retention of birds in the GM2 and FM3 groups was higher (*P* < 0.01) than those in the other groups. The values of nitrogen retention of birds were also better in grasshopper meal and fish meal based diets than in R. All the studied parameters gave lower (*P* < 0.01) results in SM3 dietary group except for FCR where reverse pattern of results were noticed. Almost a similar trend of the abovementioned parameters was also observed in the female birds (Table 5).

#### 3.3.2. Serum Chemistry Analyses

The effects of experimental diets on some blood constituents of 6-week-old male birds and female birds were presented in Tables 6 and 7, respectively. In case of both sexes, there are no treatment effects on blood parameters (*P* > 0.05) among different dietary groups.

#### 3.3.3. Leukocyte Population Distribution

There were no significant effects on leukocyte distribution in terms of neutrophil, lymphocytes, eosinophils, basophils, and monocytes in the blood of selected birds of different dietary groups (*P* > 0.05) (Table 8).

#### 3.3.4. Proximate and Mineral Composition of Meat

The effects of different diets on the proximate composition and content of some minerals of quail meat are depicted in Tables 9 and 10, respectively. The values of the abovementioned parameters in the meat of birds fed with selected diet were not influenced by dietary treatment in case of both males and females except fat content which was low (*P* < 0.01) in SM1 and SM2 dietary groups and high in FM2 and FM3 dietary groups.

### 3.4. Experiment 2

#### 3.4.1. Egg Laying Performance (Table 11)

Attainment of sexual maturity (age at first egg laid) ranged from 43 days in GM3 group to 67 days in SM3 group with lower values in grasshopper meal based diets and FM3 dietary group. The result showed that birds fed on three diets of soybean meal reached laying age much later than the other dietary groups. Weight of 1st egg laid was the highest (*P* < 0.01) in GM1 group, followed by GM3, FM3, GM2, and FM1 group, and the lowest in SM3 group. It was markedly higher in R group than soybean meal based diets but lower than grasshopper and fish meal based diets. Weight gain in egg layers was not significant among the dietary groups. However, higher mean value of weight gain was observed in GM2 and FM3 groups amounting to 12.5 g; likewise, lower mean value of weight gain was observed in birds fed on SM3 diet (6.57 g). Daily feed intake per bird during laying period was higher (*P* < 0.01) in R group, followed by FM2 and FM3. It was the least (*P* < 0.05) in birds fed on SM1 and SM3 diets, followed by increased value in grasshopper meal based diets and SM2 dietary group. Hen day production percentage was higher (*P* < 0.01) in GM2 group, followed by GM1 and FM3 groups, and then in GM3, FM1, FM2, SM1, SM2, and R groups. It was lower in SM3 group. So it was confirmed that the R group had lower 1st egg weight, weight gain, percentage of hen day production, higher feed intake, and age at 1st laid egg than the grasshopper meal and fish meal based diets.

#### 3.4.2. External Egg Quality Trait

In egg quality parameters, shell thickness of birds fed with different diets was not significantly (*P* = 0.26) affected by different dietary treatments. Egg weight was higher (*P* < 0.01) in GM1 group and then gradually followed by FM3, dietary groups of GM2, GM3, FM1, and FM2, and then R. So R group had lower egg weight than the grasshopper meal and fish meal based dietary group. Birds fed SM3 diet had the least values in terms of egg weight, egg length, and egg breadth. The GM2 fed birds had the highest values in terms of egg length (*P* < 0.01), egg breadth (*P* < 0.01), egg shape index (*P* < 0.01), and egg shell weight (*P* < 0.01). In the rest of the other dietary groups, egg shape index and egg shell weight varied insignificantly. Egg length and egg breadth were lower in R group than grasshopper meal and fish meal based diets except 50 g/kg fish meal diet, which did not vary significantly with R group. No shell-less egg was found in grasshopper meal and fish meal based dietary group and SM2 group. Among the rest of the three dietary groups, R showed higher number of shell-less eggs.

#### 3.4.3. Internal Egg Quality Trait

No significant effect was detected in the yolk (*P* = 0.1) and albumen (*P* = 0.06) content of all the dietary groups. GM2 group showed higher value in shell content (*P* < 0.01). R group had lower shell content than grasshopper meal based dietary group and higher than fish meal based dietary group.

#### 3.4.4. Chemical Composition of Edible Parts

It was notable that there were no significant effects (*P* > 0.05) among dietary treatments in the chemical composition of edible parts of egg as shown in Table 12.

#### 3.4.5. Dressed Carcasses and Body Organs

The effects of different diets on the relative weight of dressed carcass, edible inner organs, and the relative weight and length of gut at 16 weeks of age are presented in Table 13. The weights of dressed carcass of male birds fed with GM2 and FM3 diets were higher (*P* < 0.01), followed by GM3, FM2, and R group. In females, lower (*P* < 0.01) dressed carcass weight was observed in R group than in GM2, GM3, FM2, and FM3 groups.

No significant variation was revealed in DMRT among the last four groups where data were higher than the other dietary groups. ANOVA results did not show any significant difference among the birds fed on different formulated diets, in terms of relative weight of skin (male: *P* = 0.06; female: *P* = 0.14), abdominal fat (male: *P* = 1; female: *P* = 1), liver (male: *P* = 0.30; female: *P* = 0.29), heart (male: *P* = 1; female: *P* = 0.97), pancreas (male: *P* = 0.99; female: *P* = 0.97), spleen (male: *P* = 1; female: *P* = 1), and caecum length (male: *P* = 0.93; female: *P* = 0.73) whereas the mean value of gizzard weight of birds fed on GM2 diet was lower than those fed on other diets, though dietary treatment had no significant effect (male: *P* = 0.01; female: *P* = 0.01) on percent gizzard for both male and female birds. The weight of total meat of both males and females was higher in GM2, GM3, and FM3 (male: *P* < 0.01; female: *P* < 0.01) and lower in SM1 and SM3 diet fed sets and for the rest of the other dietary groups no significant difference was observed. Intestine length in female birds was significantly higher in FM3 dietary group and in the rest of the groups formulated diets had no effect on intestine length. Similarly male birds did not show any significant effect on intestine length.

In this study the reproductive organs of 16-week-old birds were influenced (*P* < 0.01) by dietary treatment (Table 14). Ovary weight was high (*P* < 0.01) in GM2 and FM3 fed group compared to the other diets fed birds where DMRT did not show any significant variation. Higher values of testes length (*P* < 0.01) and breadth (*P* < 0.01) were observed in FM3 dietary group whereas higher (*P* < 0.01) value of testes density was found in GM2 dietary group. So it was noticed that R group had lower reproductive organ proportion and weight than grasshopper meal and higher than soybean meal base diets.

## 4. Discussion 

### 4.1. Nutrient and Antinutrient Composition of Acridid Meal

Proximate composition showed that the crude protein content of the grasshopper meal was higher than that reported for many other grasshopper species such as 654 g/kg in* Acrida cinerea*, 498.7 g/kg found in* Zonocerus variegatus*, and 221.2 g/kg in grasshoppers of Nigeria [13, 27, 28]. Protein content of* O. hyla hyla* was also higher than many other insects such as from 494 g/kg to 581 g/kg in six Lepidoptera larvae, 580 g/kg found in Mormon cricket, and 620 g/kg in house cricket [6, 12, 29]. Out of 20 total amino acids required by quail, 13 are considered as essential amino acids which are arginine, glycine, histidine, isoleucine, leucine, lysine, methionine, cysteine, phenylalanine, tyrosine, threonine, tryptophan, and valine. All the essential amino acids were present in good amounts in grasshopper meal. Experiments on other insects revealed that lysine and threonine are limiting amino acids in Mormon cricket; house crickets were deficient in methionine [29]. In addition essential amino acids of six species of Lepidoptera were deficient in methionine, cysteine, and possibly lysine [6]. Thus, the studied grasshopper species had an advantage in amino acid composition compared to the other insects reported. The major lacunae of grasshopper proteins were found to be marked deficiency in tryptophan. Thus, the insect would be unsatisfactory as the sole source of dietary nitrogen for poultry, due to the limiting amino acid, but could be extremely beneficial as a complement to poultry diets [13].

The essential fatty acids are linoleic acid, linolenic acid, and oleic acid for quails. These fatty acids were found in higher amount in grasshopper meal. The level of unsaturated fatty acids in the studied acridid oil was higher than many of the animal lipids indicating that the grasshopper meal should be given some attention as a potential high-quality oil source for the feed industry [30]. According to DeFoliart, insect fatty acids are similar to that of fish in their degree of unsaturation [31]. Nutritionally, a high level of saturated fatty acids in foods might be undesirable because of the linkage between saturated fatty acids and atherosclerotic disorders. The presence of the essential fatty acids such as linoleic and linolenic in substantial amounts further points to the nutritional value of the selected acridid.

Studied antinutrient factors were present in very low amount. These values were lower than the previously studied values of some insects [32, 33].

Results of proximate composition, amino acids, fatty acids, vitamins, minerals, and antinutrient factors ensure that grasshopper meal is a suitable alternative food supplement for using formulated diets of Japanese quail.

### 4.2. Experimental Diets

According to Whyte et al., a dietary protein level of 180 to 240 g/kg was adequate for good performance of Japanese quail [34]. So the formulated diets for quail having 230 g/kg crude protein could provide adequate protein. According to Yamany et al., 7 to 8% of lipid in poultry diets gives optimum growth rates without producing an excessive fatty carcass [35]. In this context it may seem that the grasshopper meal based diets of the present study contain a little higher amount of fat (near about 8 to 9%). However, report of Odunsi et al. states that Japanese quails are fed with diets having 4–7% of fat [36]. This report supports the view that the fat contents of formulated diets (other than grasshopper meal based diets) of the present study were found in these ranges. Metabolizable energy (ME) is another important factor like protein content of diets. According to some workers for achieving maximum growth, ME in Japanese quail diet should be nearly 12 MJ/kg [37–40]. These reports are encouraging because in the present case all of the formulated diets had ME value of nearly 12 MJ/kg. Therefore, all the formulated diets are similar with respect to nutritional quality except fat contents, and they fulfil the optimum need of Japanese quail. During the feeding trial it was observed that all the experimental diets were acceptable to the Japanese quail.

### 4.3. Experiment 1

#### 4.3.1. Growth Performance

Daily weight gain in Japanese quail ranged between 3 to 4 g. This goes in concert with our findings except only SM1 and SM3 fed sets where a little lower values were obtained [39, 40]. However, the lower values are also resembling the results of the previous works of Odunsi et al. and Ayasan et al. [36, 41]. The values of FCR of the present feeding trial were similar to the results reported by Attia et al. and Ayasan et al. who opined that the value of FCR should range between 3.44 and 5.23 for Japanese quail [37, 41]. Grasshopper based diet with 100 g/kg grasshopper meal showed acceptable results of body weight gain, food consumption, and growth parameters followed by fish diet with 150 g/kg fish meal. Though food consumption was lower in GM2 than in three formulated fish diets, live body weight gain gave higher result and less amount of fecal matter with lower percentage of nitrogen was noticed in GM2. Therefore, growth parameters and nitrogen retention in the body of quail fed with GM2 diet gave preferable results.

#### 4.3.2. Serum Chemistry Analyses and Leukocyte Population Distribution

According to Fasuyi et al., during feeding experiments haematological examination is essential for assessment of clinical and nutritional health of the animal in question [1]. A similar trend obtained for serum metabolites is comparable with many other reports [35, 42]. All the birds were in normal physiological status as their blood serum biochemical constituents and leukocyte distribution were more or less equal and insignificantly varied.

#### 4.3.3. Mineral Composition of Meat

The value of poultry meat is determined by its nutrient composition. The range of mean dry matter contents of quail meat of dietary group was similar to those earlier reports but lower than that given by Christaki et al. and these differences could be partially explained by the diet formulation, breed, and age of the quails [43–45].

The nutritive and dietary values of meat mainly depend on the protein and fat it contains. The range of fat content confirms the result of the earlier workers [44, 46]. According to Yalçin and Oğuz, these differences occurred due to either the content of carcasses, such as feather, bone, skin, and abdominal fat, or breed, sex, and diet formulation that could have significant effects on the fat content of the carcass [44]. Additionally, Japanese quail is an active bird and its fat content remains very low until it reaches maturity.

Mean protein content range of carcass meat in this study was similar to that reported by Yalcin et al. and Sunder et al. [44, 47] but higher than that given by Marks [48] and lower than those in some other studies [45, 49]. The protein content of carcass meat was not influenced by dietary treatment. This opinion was also supported by Yalçin and Oğuz [44].

### 4.4. Experiment 2

#### 4.4.1. Egg Laying Performance

Wakasugi reported that, in Japanese quail, onset of egg laying was between 42 and 50 days of age [50]. A similar trend was observed in case of grasshopper meal and fish meal based dietary groups. The similar values across the dietary treatments during laying period in terms of performance, that is, weight gain, might be due to the ability of quails to utilize the nutrients and were nearly the same. This observation was supported by Odunsi et al. and NVRI [36, 51]. It is of interest to note that any gain in body weight after commencement of egg production was minimal as the bird was essentially at its mature body weight.

#### 4.4.2. External Egg Quality Trait

Egg production and egg quality traits conformed and compared favourably (even higher in some cases) with values reported for layers in available literatures [36, 37, 52]. In egg quality parameters, egg weight of birds fed with the selected diets did not decline by the increase of egg production in different dietary groups during experimental period. These results are in good agreement with that of Abdel-Azeem et al., who stated that egg production, egg weight, and egg mass were improved in laying hens fed on diets containing proper nutrients [53]. Experimental diets have significant effects on feed intake, egg production, egg shell weight, egg shape index, and number of eggs, but no significant variation was obtained in case of egg shell thickness as found in previous work [41].

Effect of experimental feeds on laying performance and egg quality values of Japanese quails gave better results in birds fed with 50 g/kg grasshopper meal diet than 50 g/kg fish meal diet, 100 g/kg grasshopper meal diet than 100 g/kg fish meal diet, and 150 g/kg fish meal diet than 150 g/kg grasshopper meal diet. Additionally, both the grasshopper meal and fish meal added diet fed groups showed better results than the R group. Among all the groups of grasshopper meal and fish meal based diets, 100 g/kg grasshopper meal based diet gave more acceptable results, followed by 150 g/kg fish meal based dietary group.

#### 4.4.3. Internal Egg Quality Trait

According to Tarasewicz et al., the most favourable albumen to yolk ratio was 1.88 : 1. In this study, similar data was found in grasshopper based diets [52].

#### 4.4.4. Dressed Carcasses and Body Organs

Higher mean value of intestine length of FM3 group might be due to their higher amount of feed intake. Significantly higher percentages of dressed carcass and total meat in quails fed with both the GM2 and FM3 diets reflect that both diets are suitable for the quails. Higher percentage of ovary weight and testes density might be due to higher reproductive potential of GM2 group.

Though carcass percentages and total meat percentages were similar in 100 g/kg grasshopper meal and 150 g/kg fish meal dietary groups, 100 g/kg grasshopper meal diet (GM2) was more suitable feed for quails as other studied parameters gave better results in this case and consumption of GM2 diet per bird was significantly lower than 150 g/kg FM3 diet during both growing period and laying period. Birds fed with different formulated diets have no significant effects on their organ proportions which indicates that the grasshopper meal based diets used in this experiment are not in any way harmful to the quails. Ojewola et al. agreed with this finding and stated that satisfactory nonconventional animal protein could be prepared from grasshopper meal [54]. Nonoccurrence of mortality among the birds fed on grasshopper meal diets might also be an indication of the safety of grasshopper meal as a suitable alternative feed supplement for quail birds.

## 5. Conclusion

The present study revealed that* O. hyla hyla* has the potential to provide substantial amounts of protein, minerals, vitamins, amino acids, and fatty acids to the diet of formulated feed of Japanese quail. Formulated diet having* O. hyla hyla* meal added in the proportion of 100 g/kg had no adverse effects as feedstuff; also they showed pronounced positive response on Japanese quail as most suitable, alternative feedstuff. Moreover, the growth performance and laying performance of Japanese quail were better in most of the cases in 100 g/kg grasshopper meal diet followed by 150 g/kg fish meal based diets. Based on this fact, it could be safely recommended that quails could be easily fed on 100 g/kg grasshopper meal diet without deleterious effect on growth performance, physiological parameters, and egg quality parameters.

## Figures and Tables

**Table 1:** Amino acid content of grasshopper meal, fish meal, and soybean meal.

Properties	Grasshopper meal		Fish meal		Soybean meal	
Amino acids (g/kg of protein)	Mean	SD	Mean	SD	Mean	SD
Aspartic acid	28.42	0.1	44.21	4.6	6.02	0.03
Glutamic acid	25.01	0.1	58.08	5.9	6.88	0.03
Serine	47.13	0.2	20.15	0.6	2.61	0.09
Glycine	53.01	0.4	30.02	0.2	1.64	0.03
Histidine	70.00	0.4	66.96	6.3	2.52	0.01
Arginine	74.90	0.3	30.84	0.6	7.28	0.72
Threonine	204.80	4.4	19.83	0.9	1.44	0.03
Alanine	22.15	0.1	29.52	2.8	2.91	0.11
Proline	156.61	2.5	26.84	0.7	1.92	0.02
Tyrosine	50.90	0.6	17.01	0.6	3.35	0.11
Valine	30.22	0.2	25.22	0.8	4.21	0.02
Methionine	19.20	0.1	14.47	0.5	0.49	0.01
Cysteine	08.62	0.2	3.98	0.3	0.79	0.02
Isoleucine	15.12	0.1	22.83	0.8	1.88	0.01
Leucine	68.14	0.5	42.55	0.9	3.66	0.01
Phenylalanine	56.42	0.3	21.59	0.76	2.54	0.02
Lysine	69.00	0.3	44.05	1.16	6.49	0.22
Tryptophan	0.51	0.1	0.49	0.01	0.42	0.01

**Table 2:** Nutrient and antinutrient contents of *Oxya hyla hyla* (on dry matter basis).

Properties	*O. hyla hyla *	
Fatty acids (g/kg of fat)	Mean	SD
Myristic acid	11.81	0.3
Palmitic acid	10.80	1.0
Palmitoleic acid	20.10	1.0
Oleic acid	305.20	8.1
Stearic acid	10.22	0.1
Eicosenoic acid	20.20	4.2
Linoleic acid	420.51	11.2
Linolenic acid	125.72	12.0
Vitamins (mg/kg)	Mean	SD
Vitamin A (retinol)	60.92	0.4
Vitamin B1 (thiamine)	2.70	0.1
Vitamin B2 (riboflavin)	14.20	0.1
Vitamin B3 (niacin)	63.20	0.7
Vitamin C (ascorbic acid)	46.92	0.5
Minerals (mg/kg)	Mean	SD
Calcium	12.97	0.32
Magnesium	8.06	0.02
Zinc	4.20	0.08
Iron	5.18	0.07
Manganese	0.73	0.01
Copper	1.79	0.04
Antinutrients (g/kg)	Mean	SD
Oxalate	4.80	0.4
Tannin	10.21	0.3
Phytin	0.64	0.03
Phytin P	0.11	0.005

All values were the means of three determinations.

**Table 3:** Ingredients and chemical compositions of the experimental diets (on dry matter basis in g/kg).

Ingredients	GM1	GM2	GM3	FM1	FM2	FM3	SM1	SM2	SM3	R
Grasshopper meal	50.0	100.0	150.0	0.0	0.0	0.0	0.0	0.0	0.0	0.0
Fish meal	0.0	0.0	0.0	50.0	100.0	150.0	0.0	0.0	0.0	10.0
Soymeal	0.0	0.0	0.0	0.0	0.0	0.0	50.0	100.0	150.0	145.3
Maize	500.0	500.0	500.0	500.0	500.0	500.0	500.0	500.0	500.0	600.0
Ground nut cake	302.7	220.5	138.3	322.7	260.5	198.1	334.9	284.9	234.8	100.0
Rice husk	117.3	149.5	181.6	97.3	109.5	121.9	85.1	85.1	85.1	40.7
Oyster shell	16.8	17.1	17.2	13.3	13.8	14.1	12.8	13.3	13.6	0.0
DCP	7.0	7.0	7.0	7.0	7.0	7.0	7.0	7.0	7.0	3.4
Salt	2.0	2.0	2.0	2.0	2.0	2.0	2.0	2.0	2.0	2.0
Vitamin-mineral mixture^a,b^	3.5	3.5	3.5	3.5	3.5	3.5	3.5	3.5	3.5	3.5
DL-Met	0.5	0.2	0.2	2.7	2.5	2.3	2.8	2.5	2.4	2.8
DL-Lys	0.3	0.2	0.1	1.5	1.3	1.1	1.9	1.7	1.5	1.8

Total	1000	1000	1000	1000	1000	1000	1000	1000	1000	1000

Proximate composition (g/kg)										
CP	231.0	233.0	232.0	232.0	231.0	230.0	230.0	230.0	232.0	230.0
CL	67.0	64.5	62.5	68.0	65.2	61.8	67.5	65.1	61.2	65.1

Digestible Lys	11.7	11.7	11.7	11.7	11.7	11.7	11.7	11.0	11.7	11.7
Digestible Met	4.1	4.1	4.1	4.1	4.0	4.0	4.1	4.1	4.1	4.1
Digestible Met + Cys	9.2	9.2	9.2	9.2	9.2	9.2	9.2	9.2	9.2	9.2
Metabolizable energy (MJ/kg)	12.2	12.2	12.3	12.2	12.2	12.3	12.2	12.1	12.1	12.1
Nonphytate P	5.9	5.9	5.9	5.9	5.9	5.9	5.9	5.9	5.9	5.9

^a^Provided per kg of diet of growing quail: Vitamin A: 9500 IU; Vitamin D3: 3050 IU; Vitamin E: 20 mg; thiamine: 1 mg; riboflavin: 6.5 mg; pyridoxine: 2 mg; biotin: 0.05 mg; folic acid: 0.30 mg; Ca-pantothenate: 10 mg; Fe: 54 mg; Zn: 50 mg; Cu: 5 mg; Mn: 80 mg; I: 1 mg; Se: 0.2 mg; Sand: 3.26.

^b^Provided per kg of diet of laying quails: Vitamin A: 9500 IU; Vitamin D3: 3050 IU; Vitamin E: 10 mg; thiamine: 1 mg; riboflavin: 6.5 mg; pyridoxine: 2 mg; biotin: 0.05 mg; folic acid: 0.30 mg; Ca-pantothenate: 3.28 g; Fe: 54 mg; Zn: 50 mg; Cu: 5 mg; Mn: 80 mg; I: 1 mg; Se: 0.2 mg.

**Table 4:** Effect of experimental feed on body weight gain, food consumption, and growth indices of male quail on the basis of single individual.

Parameter	Diets										Pooled SEM
	GM1	GM2	GM3	FM1	FM2	FM3	SM1	SM2	SM3	R	
Initial weight (g)	21.20a	21.70a	21.20a	21.20a	21.40a	21.70a	21.50a	21.50a	21.40a	21.30a	0.534
Weight gain (g/bird/day)	3.36d	4.04f	3.59d	3.24c	3.47d	3.72e	2.79b	3.13c	2.63a	3.05c	0.006
Feed intake (g/bird/day)	13.69c	13.40c	13.79c	14.00c	14.62d	15.13e	12.54b	13.14c	12.27a	13.31c	0.014
FCR^a^	4.07c	3.33a	3.84b	4.32d	4.21d	4.07c	4.50d	4.21d	4.67e	4.37d	0.013
PER^b^	1.07b	1.30d	1.13c	1.01b	1.03b	1.07b	0.97b	1.03b	0.93a	1.00b	0.001
N retention (g/bird/day)	1.84c	2.05e	1.96d	1.78c	1.90c	2.03e	1.59b	1.73c	1.44a	1.66c	0.002

Mean ± SD; *n* = 3. Within a row, a, b, c, d, e, and f indicate significant differences between mean values. (One-way ANOVA; DMRT; *P* < 0.01).

^a^Feed conversion ratio.

^b^Protein efficiency ratio.

**Table 5:** Effect of experimental feed on body weight gain, food consumption, and growth indices of female quail on the basis of single individual.

Parameters	Diets										Pooled SEM
	GM1	GM2	GM3	FM1	FM2	FM3	SM1	SM2	SM3	R	
Initial weight (g)	24.50a	24.70a	24.70a	24.30a	24.20a	24.20a	24.50a	24.40a	24.50a	24.30a	0.363
Weight gain (g/bird/day)	3.74c	5.01g	4.11e	3.60c	3.91d	4.40f	2.99b	3.42c	2.71a	3.23c	0.008
Feed intake (g/bird/day)	15.14b	14.86b	15.34b	15.55b	16.20c	16.64d	13.98a	14.54b	13.85a	14.72b	0.013
FCR^a^	4.05c	2.97a	3.74b	4.32c	4.14c	3.78b	4.68d	4.25c	5.11e	4.56d	0.015
PER^b^	1.07c	1.46e	1.16d	1.00c	1.05c	1.15d	0.93b	1.02c	0.85a	0.95b	0.001
N retention (g/bird/day)	1.89c	2.08d	1.99c	1.85c	1.96c	2.07d	1.60a	1.75b	1.49a	1.71b	0.003

Mean ± SD; *n* = 3. Within a row, a, b, c, d, e, f and g indicate significant differences between mean values. (One-way ANOVA; DMRT; *P* < 0.01).

^a^Feed conversion ratio.

^b^Protein efficiency ratio.

**Table 6:** Effect of experimental diet on biochemical constituents of blood serum from 6-week-old male quail on the basis of single individual.

Criteria	Diets										Pooled SEM
	GM1	GM2	GM3	FM1	FM2	FM3	SM1	SM2	SM3	R	
Total protein (g/L)	31.5	33.2	30.3	30.3	30.8	30.6	29.2	30.5	26.8	29.7	9.7
Albumin (g/L)	13.2	13.4	13.1	13.2	13.0	13.5	12.9	13.3	13.2	13.1	0.1
AST (U/L)	430.7	443.0	443.3	430.7	427.7	431.3	424.3	442.0	421.3	440.3	299.5
ALT (U/L)	10.2	10.2	10.2	9.9	10.1	10.1	10.0	9.9	10.1	10.1	0.6
Glucose (mg/dL)	132.1	146.9	141.5	129.0	143.1	125.6	14.2	125.9	118.4	130.5	38.8
Cholesterol (mg/dL)	203.9	203.6	203.7	202.5	199.3	203.0	205.3	204.0	197.3	203.0	25.3
Triglyceride (mg/dL)	127.5	129.8	120.6	128.8	122.4	126.5	111.1	117.1	112.4	118.5	43.8
HDL (mg/dL)	87.7	87.4	94.6	81.5	86.4	92.4	81.5	80.4	80.3	87.3	7.0
LDL (mg/dL)	5.1	5.1	5.0	4.9	4.9	4.9	5.0	5.0	5.0	5.1	0.1
VLDL (mg/dL)	113.4	115.8	108.5	115.2	110.0	113.8	99.0	105.1	101.0	106.2	48.1
Bilirubin (*µ*ml/L)	20.9	22.5	22.2	22.2	22.7	23.5	23.4	23.5	23.1	22.2	2.7
Creatinine (*µ*mol/L)	3.9	4.0	3.8	4.0	3.9	3.9	4.0	3.9	3.9	4.0	0.4
Calcium (mg/dL)	10.7	10.6	10.6	10.6	10.4	10.3	10.5	10.4	10.4	10.3	0.1
Phosphorus (mg/dL)	5.6	5.7	5.5	5.4	5.3	5.3	5.3	5.1	5.2	5.4	0.4

Mean ± SD; *n* = 3. No significant effect was found among the different dietary groups. (One-way ANOVA; *P* > 0.01).

**Table 7:** Effect of experimental diet on biochemical constituents of blood serum from 6-week-old female quail on the basis of single individual.

Criteria	Diets										Pooled SEM
	GM1	GM2	GM3	FM1	FM2	FM3	SM1	SM2	SM3	R	
Total protein (g/L)	35.4	36.5	35.8	35.6	35.3	36.6	35.2	33.8	33.4	34.7	4.7
Albumin (g/L)	15.3	15.4	15.2	15.3	15.2	15.2	14.9	15.1	15.2	15.4	0.2
AST (U/L)	362.0	370.0	362.3	364.4	355.9	365.1	365.2	362.8	352.4	366.0	185.0
ALT (U/L)	7.1	7.2	6.2	7.2	7.1	7.0	7.1	7.1	7.1	7.1	2.3
Glucose (mg/dL)	124.3	133.6	133.3	124.8	132.0	126.5	115.1	121.5	114.3	125.5	76.8
Cholesterol (mg/dL)	209.1	217.3	218.3	215.2	217.3	216.0	209.7	211.3	210.5	216.3	21.1
Triglyceride (mg/dL)	174.6	163.9	174.3	184.2	170.0	182.0	173.0	166.0	180.9	178.2	18.7
HDL (mg/dL)	39.1	36.7	39.2	41.4	37.8	40.9	38.8	37.3	40.7	40.0	0.9
LDL (mg/dL)	6.3	6.2	6.2	6.2	6.3	6.1	6.2	6.2	6.3	6.2	0.1
VLDL (mg/dL)	156.5	147.2	156.8	165.7	152.9	163.6	155.8	149.1	162.8	160.1	85.8
Bilirubin (*µ*ml/L)	10.8	10.9	10.9	10.8	10.9	10.9	11.1	11.0	11.0	11.1	0.6
Creatinine (*µ*mol/L)	5.1	4.9	5.0	5.0	5.0	5.0	5.1	5.0	4.9	5.0	0.1
Calcium (mg/dL)	11.2	11.5	11.1	11.2	11.4	11.2	11.2	11.3	10.9	11.2	0.2
Phosphorus (mg/dL)	6.3	6.5	6.4	6.2	6.4	6.3	6.3	6.1	6.1	6.1	0.7

Mean ± SD; *n* = 3. No significant effect was found among the different dietary groups. (One-way ANOVA; *P* > 0.01).

**Table 8:** Leukocyte population distribution from 6-week-old quail on the basis of single individual.

Leukocyte type	Sex	Diets										Pooled SEM
		GM1	GM2	GM3	FM1	FM2	FM3	SM1	SM2	SM3	R	
Neutrophil	Male	19.3	19.0	18.7	19.0	19.7	20.3	19.7	19.7	20.3	19.3	6.1
	Female	20.7	21.0	20.7	20.7	20.7	21.0	20.7	21.0	20.7	21.0	2.6
Lymphocytes	Male	62.0	61.3	60.7	61.3	60.7	61.3	62.0	62.3	61.7	61.7	3.1
	Female	66.0	66.7	66.7	67.3	67.0	67.7	68.3	67.0	67.3	67.3	3.2
Eosinophils	Male	3.7	4.0	3.7	3.7	3.7	4.0	4.0	3.7	3.7	4.0	1.2
	Female	3.3	3.3	3.3	3.3	3.3	3.3	3.3	3.3	3.3	3.3	1.3
Basophils	Male	1.3	1.3	1.0	1.3	1.3	1.0	1.0	1.0	1.3	1.3	0.4
	Female	0.7	0.7	0.7	1.0	1.0	0.7	1.0	1.0	0.7	1.0	0.3
Monocytes	Male	13.7	13.0	13.3	13.7	13.7	13.7	13.3	13.3	13.7	13.7	2.8
	Female	7.7	8.3	8.3	8.7	8.7	8.7	8.3	8.3	8.7	7.7	1.3

Mean ± SD; *n* = 3. No significant effect was found among the different dietary groups. (One-way ANOVA; *P* > 0.01).

**Table 9:** Effects of experimental diets on proximate composition of 6-week-old quail meat.

Criteria (g/kg)	Sex	Diets										Pooled SEM
		GM1	GM2	GM3	FM1	FM2	FM3	SM1	SM2	SM3	R	
Dry matter	M	246.8	256.0	245.0	245.0	249.4	250.1	248.0	256.4	245.5	248.3	19.1
	F	247.0	256.0	246.0	244.9	250.6	249.8	252.0	254.0	250.1	242.1	38.3
Protein	M	199.6	202.0	198.0	195.0	201.2	201.7	184.0	192.5	181.3	193.0	5.3
	F	200.0	203.0	199.0	196.0	201.8	202.5	183.0	192.8	181.7	193.7	12.0
Fat	M	44.4b	42.9b	45.1b	44.3b	56.4c	57.0c	26.7a	44.4b	25.4a	43.8b	20.2
	F	44.5b	41.2b	49.2b	54.7c	59.2c	51.0c	36.1a	47.3b	34.1a	46.9b	15.6
Ash	M	28.7	30.5	30.0	29.8	30.3	30.2	28.9	29.3	28.4	29.4	3.1
	F	26.6	27.8	27.2	27.0	27.7	27.4	27.8	27.6	27.2	26.9	13.3
Energy (MJ/kg)	M	5.3	5.4	5.2	4.9	5.3	5.3	5.0	5.2	4.9	5.2	0.0
	F	5.7	5.7	5.7	5.7	5.7	5.7	5.3	5.6	5.2	5.6	0.0

Mean ± SD; *n* = 3. Within a row, a, b and c indicate significant differences between mean values. (One-way ANOVA; DMRT; *P* < 0.01).

**Table 10:** Effects of experimental diets on mineral content of 6-week-old quail meat.

Criteria (g/kg)	Sex	Diets										Pooled SEM
		GM1	GM2	GM3	FM1	FM2	FM3	SM1	SM2	SM3	R	
Ca	M	18.7	19.6	17.9	17.5	18.6	19.0	14.8	16.6	15.3	16.7	0.4
	F	20.7	22.1	20.6	20.4	20.4	21.9	16.5	18.8	16.0	19.3	0.5
Mg	M	27.7	27.7	27.6	27.6	27.6	27.6	27.6	27.4	27.4	27.6	13.8
	F	27.6	27.7	27.6	27.6	27.4	27.6	27.5	27.3	27.4	27.4	0.2
P	M	181.7	178.0	180.3	178.0	172.7	176.3	170.0	172.0	170.3	170.0	254.2
	F	146.0	147.0	143.3	143.7	144.3	141.7	142.3	148.0	144.0	145.0	56.4
Fe	M	2.0	2.1	2.0	2.0	1.9	1.9	1.9	1.8	1.9	2.0	0.3
	F	1.8	1.8	1.7	1.7	1.7	1.7	1.6	1.7	1.6	1.7	0.0
Zn	M	1.1	1.1	1.1	1.0	1.0	1.1	1.0	1.0	1.0	1.1	0.0
	F	1.7	1.7	1.6	1.6	1.6	1.6	1.6	1.6	1.5	1.7	0.0
K	M	342.3	340.0	342.0	342.7	345.7	345.3	343.3	340.0	339.7	338.0	20.2
	F	324.7	323.7	322.3	322.7	325.3	326.3	322.7	322.3	321.3	322.3	14.7

Mean ± SD; *n* = 3. No significant effect was found among the different dietary groups. (One-way ANOVA; *P* > 0.01).

**Table 11:** Effect of experimental feeds on laying performance and egg quality values of quails on the basis of single individual.

Criteria	Diets										Pooled SEM
	GM1	GM2	GM3	FM1	FM2	FM3	SM1	SM2	SM3	R	
Laying period											
Age at 1st egg (day)	51.6a	50.0a	43.0a	56.7b	54.3b	48.0a	63.7d	61.7c	67.3e	58.0b	4.8
Weight of 1st egg laid (g)	11.7f	10.2d	10.8e	9.8d	9.3c	11.2e	8.2b	7.4a	7.0a	9.0c	2.7
Weight gain (g)	11.8a	12.5a	11.2a	10.1a	9.5a	12.5a	7.5a	8.4a	6.6a	8.2a	11.2
% hen day production	62.2c	72.2d	49.5b	44.8b	52.3b	63.5c	39.4b	46.4b	31.9a	41.8b	13.4
Feed intake (g/day/bird)	26.4b	25.1b	27.6b	29.7c	32.2d	31.9d	23.7a	27.1b	22.9a	33.6e	0.8

Egg quality											
Shell thickness (mm)	0.2	0.3	0.3	0.2	0.2	0.3	0.2	0.2	0.2	0.2	0.002
Egg weight (g)	12.8i	11.7g	11.8g	10.9f	10.6e	12.5h	9.8c	8.7b	7.6a	10.3d	0.003
Egg length (cm)	2.8c	3.0e	2.9d	2.6b	2.7c	3.0d	2.6b	2.5b	1.8a	2.6b	0.002
Egg breadth (cm)	2.2c	2.5e	2.3c	2.1b	2.2c	2.4d	2.0b	1.9b	1.5a	2.0b	0.001
Egg shape index	0.80a	0.83b	0.80a	0.78a	0.79a	0.81a	0.78a	0.76a	0.79a	0.78a	0.0
Egg shell weight (g)	0.7a	0.8b	0.7a	0.7a	0.6a	0.7a	0.6a	0.7a	0.6a	0.6a	0.0
Shell-less egg %	0.0a	0.0a	0.0a	0.0a	0.0a	0.0a	1.0b	0.0a	3.0b	9.0c	0.6

Egg proportions (g/kg)											
Yolk	303.0	302.4	314.8	298.8	300.0	302.4	296.8	284.0	287.0	309.6	1180.0
Albumen	618.0	618.6	603.1	628.2	627.0	624.2	636.8	646.8	644.2	616.2	2124.0
Shell	75.2f	78.5h	76.2g	71.5c	72.9d	73.2d	66.22a	69.1b	68.7b	73.9e	0.1

Mean ± SD; *n* = 3. Within a row, a, b, c, d, e, f, g, h and i indicate significant differences between mean values. (One-way ANOVA; DMRT; *P* < 0.01).

**Table 12:** Chemical compositions (g/kg) of the edible parts of egg of quail.

Criteria	Diets										Pooled SEM
	GM1	GM2	GM3	FM1	FM2	FM3	SM1	SM2	SM3	R	
Egg yolks (g/kg)											
Dry matter	524.1	524.9	525.7	524.9	523.5	524.7	522.5	525.5	521.2	524.8	8.7
Crude protein	295.2	296.1	295.4	295.4	295.8	295.4	295.2	294.1	294.0	296.0	36.1
Ether extract	629.5	635.0	632.8	631.1	633.8	633.0	627.9	629.6	627.7	630.5	119.8

Egg albumen (g/kg)											
Dry matter	118.4	119.7	118.3	118.2	118.3	118.2	116.9	118.9	116.4	115.8	3.5
Crude protein	840.9	840.2	840.7	840.7	840.3	840.9	840.4	840.7	840.1	840.7	22.6
Ether extract	86.0	87.0	87.1	87.0	86.9	87.0	86.9	87.1	86.8	87.2	1.0

Edible parts (g/kg)											
Dry matter	262.3	260.9	260.5	259.7	256.9	260.1	259.9	259.8	258.7	258.8	9.7
Crude protein	475.1	476.4	475.7	475.6	476.2	475.9	472.9	474.6	471.9	475.2	40.8
Ether extract	465.3	465.0	464.2	465.2	464.5	463.8	464.0	463.1	463.5	464.1	36.2

Mean ± SD; *n* = 3. No significant effect was found among the different dietary groups. (One-way ANOVA; *P* > 0.01).

**Table 13:** Effect of experimental feeds on carcasses characteristics and organ proportions of 16-week-old quail on the basis of single individual.

Criteria (g/kg body weight)		Diets										Pooled SEM
		GM1	GM2	GM3	FM1	FM2	FM3	SM1	SM2	SM3	R	
Carcass	M	580.5c	714.4e	640.5d	534.4b	617.5d	678.5e	492.5a	526.5b	449.2a	577.7c	911.9
	F	633.5c	727.2d	670.5d	553.1b	652.4d	683.9d	510.8a	553.7b	478.5a	603.5c	841.1
Skin	M	0.2	0.1	0.1	0.1	0.2	0.2	0.1	0.2	0.1	0.1	0.0
	F	0.2	0.1	0.1	0.2	0.2	0.2	0.1	0.2	0.1	0.1	0.0
Abdominal fat	M	11.5a	11.7a	11.7a	11.5a	11.7a	11.9b	11.5a	11.3a	11.2a	11.5a	1.7
	F	11.7a	11.6a	11.7a	11.6a	11.6a	11.8b	11.9a	11.6a	11.3a	11.4a	1.7
Total meat	M	322.1b	361.6c	343.9c	301.8b	315.5b	365.2c	269.8a	286.6b	261.5a	290.2b	243.3
	F	335.2b	376.5c	368.3c	315.8b	330.8b	371.9c	261.6a	321.7b	250.3a	330.3b	183.7
Liver	M	20.1	24.1	24.5	20.1	24.1	26.5	24.6	24.5	26.3	24.6	22.3
	F	20.7	24.6	26.5	20.5	24.1	27.9	26.3	25.9	26.5	24.7	27.9
Heart	M	8.5	8.5	8.4	8.3	8.5	8.6	8.5	8.4	8.6	8.4	1.1
	F	8.4	8.6	8.2	8.1	8.5	8.5	8.6	8.6	8.5	8.4	0.6
Gizzard	M	23.0	19.6	23.5	22.6	21.9	25.4	25.8	27.7	25.2	23.2	9.2
	F	22.6	19.9	23.9	22.4	21.6	26.0	26.0	27.6	25.4	23.0	10.5
Pancreas	M	3.0	2.9	2.9	2.9	2.9	2.9	2.8	2.8	2.8	2.9	0.1
	F	3.0	2.9	2.8	3.0	2.9	2.9	2.8	2.9	2.9	2.8	0.2
Spleen	M	1.4	1.3	1.4	1.4	1.4	1.4	1.4	1.4	01.4	1.4	3.5
	F	1.4	1.4	1.4	1.4	1.4	1.4	1.4	1.4	1.5	1.4	0.1
Intestine length (cm/kg body weight)	M	260.5	242.2	224.0	192.5	261.2	273.8	183.5	201.5	180.9	254.5	335.3
	F	262.8a	257.1a	236.1a	197.3a	274.8a	297.0b	198.5a	211.6a	18.8a	262.3a	311.0
Ceacum length % (cm/kg body weight)	M	100.5	104.0	95.1	94.1	102.5	95.0	87.5	102.5	93.0	100.5	453.4
	F	102.5	104.4	94.7	92.9	103.1	89.6	87.0	103.7	89.6	103.8	447.5

Mean ± SD; *n* = 3. Within a row, a, b, c, d, and e indicate significant differences between mean values. (One-way ANOVA; DMRT; *P* < 0.01).

**Table 14:** Effect of experimental feeds on ovary and testis of 16-week-old quail on the basis of single individual.

Criteria	Diets										Pooled SEM
	GM1	GM2	GM3	FM1	FM2	FM3	SM1	SM2	SM3	R	
Ovary weight (g/kg body weight)	9.0a	11.4b	10.4a	7.6a	8.3a	16.4b	5.1a	6.1a	5.0a	6.9a	2.1
Testis length (cm)	2.4d	2.6e	2.6e	2.2c	2.3d	3.0f	1.9a	2.0b	1.8a	2.1b	0.0
Testis width (cm)	1.6d	1.7e	1.8g	1.5c	1.7f	1.9h	1.2b	1.3c	1.1a	1.4c	0.0
Testis density	0.5a	0.7b	0.5a	0.5a	0.5a	0.5a	0.4a	0.4a	0.4a	0.5a	0.0

Mean ± SD; *n* = 3. Within a row, a, b, c, d, e, f, g, and h indicate significant differences between mean values. (One-way ANOVA; DMRT; *P* < 0.01).
